# TCAP links environmental cues to circadian control of the cardiac sarcomere

**DOI:** 10.1080/15592294.2026.2754556

**Published:** 2026-11-03

**Authors:** Cecilia Facchi, Paola Cattaneo

**Affiliations:** Department of Pharmacological and Biomedical Sciences, University of Milan, via Balzaretti 9, Milan 20133, Italy; Monzino Cardiology Center, Unit of Cardiac Development and Regeneration, via Carlo Parea 4, Milan 20138, Italy

Circadian rhythms allow the cardiovascular system to anticipate recurrent changes in workload, blood pressure, metabolism and neurohumoral activity across the day. At the cellular level, this temporal organization relies on the molecular clock, in which transcription factors including CLOCK and BMAL1 generate rhythmic gene-expression programs extending far beyond the canonical clock machinery [1]. Disruption of these programs is associated with cardiovascular dysfunction and disease [2], yet considerably less is known about how clock-controlled genes integrate environmental signals to shape cardiomyocyte adaptation, particularly during cardiac maturation and hypertrophic growth.

In this issue of *Epigenetics*, Arrieta and colleagues address this question by focusing on Telethonin (*Tcap*, encoding TCAP/Titin-cap) and Myosin light chain 2 (*Myl2*) asking how their temporal regulation responds to extracellular signals relevant to postnatal cardiac maturation and hypertrophy [3]. Their findings extend an emerging connection between the cardiac clock, chromatin remodeling and sarcomere organization, while raising the intriguing possibility that communication between the circadian machinery and the contractile apparatus may be bidirectional.

TCAP is particularly well positioned at this interface. Originally characterized as a component of the sarcomeric Z-disc, TCAP binds the N-terminal region of titin and contributes to the mechanical and structural functions of the sarcomere [4]. Loss of TCAP produces relatively modest cardiac phenotype at baseline but leads to maladaptive adaptation to mechanical overload [5]. Ibrahim and colleagues, for example, demonstrated that *Tcap*-deficient cardiomyocytes develop progressive abnormalities in t-tubule organization and Ca^2+^ handling, with particularly severe remodeling following mechanical overload [6]. Together, these findings support a role for TCAP in the load-sensitive machinery that couples sarcomeric organization to the mechanical environment [5,6].

Importantly, TCAP is also under circadian control. Podobed and colleagues demonstrated rhythmic oscillations of *Tcap* mRNA and TCAP protein abundance in mouse heart, persistence of *Tcap* rhythmicity in constant darkness, blunted expression in *Clock* mutant hearts and BMAL1 recruitment to the *Tcap* promoter. Functional promoter experiments further identified an E-box through which BMAL1 regulates *Tcap* promoter activity [7]. Thus, TCAP provided an early example of how the molecular clock might impose temporal organization directly upon the cardiac sarcomere.

A previous work by Arrieta and colleagues connected the cardiac circadian system to chromatin dynamics, adding an important epigenetic layer. They demonstrated that depletion of BMAL1 in neonatal cardiomyocytes decreases cell size, and transcription of cardiac clock-controlled genes, including *Tcap* while impairing replication-independent histone turnover. Depletion of the histone variant H3.3 similarly inhibited cardiomyocyte hypertrophy and clock-controlled transcription in response to α-adrenergic stimulation [8]. Together, these observations suggested that extracellular growth signals do not simply act in parallel with the circadian clock but rather, clock-dependent chromatin dynamics participate in the transcriptional response to hypertrophic stimuli.

The new study expands this concept by asking how different environmental inputs modify *Tcap* expression [3]. *Myl2* provides an informative comparison, since its transcription is also sensitive to BMAL1 depletion, although direct CLOCK/BMAL1 regulation has been more extensively established for *Tcap* [3,7]. In synchronized neonatal rat ventricular myocytes (NRVMs), α-adrenergic stimulation with phenylephrine [3] followed by β-adrenergic stimulation with isoproterenol [3] at different phases generated phase-dependent changes in cardiomyocyte size and TCAP abundance. Prior α-adrenergic stimulation also modified the subsequent transcriptional response of *Tcap* to β-adrenergic stimulation. In contrast, *Myl2* displayed a pronounced temporal pattern of nascent transcription that was comparatively resistant to adrenergic perturbation, while MYL2 protein abundance remained relatively stable [3]. Importantly, circadian regulation of the sarcomere does not appear to represent a single transcriptional program imposed uniformly upon structural genes. Rather, individual sarcomeric genes may differ in their sensitivity to environmental signals and in the relationship between transcription and protein abundance. TCAP is particularly interesting in this regard because its temporal changes in protein abundance are not simply mirrored by changes in de novo *Tcap* transcription [3]. This difference between transcription and protein abundance is consistent with the possibility that TCAP is regulated at multiple levels. Previous work in skeletal muscle identified TCAP as a substrate of the clock-controlled E3 ubiquitin ligase FBXL21 and implicated GSK-3β:FBXL21 pathway in rhythmic post-translational TCAP degradation [9]. FBXL21 interacts with TCAP in a circadian manner that is opposite to TCAP accumulation, while GSK-3β phosphorylates both FBXL21 and TCAP to promote FBXL21-mediated degradation [9]. These mechanisms were characterized primarily in skeletal muscle rather than cardiomyocytes, nevertheless, they provide a useful precedent for circadian regulation of TCAP abundance beyond transcription and raise the possibility that related post-translational mechanisms contribute to the TCAP protein dynamics observed by Arrieta and colleagues.

Arrieta and colleagues further extend the repertoire of environmental signals by examining cardiomyocyte density and response to hypoxia. Increasing cell density altered the timing and amplitude of hypertrophy and modified the kinetics of *Per2* and *Tcap* transcription. Conditioned medium from high-density cultures was sufficient to inhibit growth when transferred to lower-density cultures, supporting the existence of a transferable extracellular signal consistent with paracrine or autocrine communication. The identity of this signal remains unknown, but the observation raises the possibility that cardiomyocyte biomass itself contributes to the temporal regulation of cardiac growth. The effects of oxygen tension provide a particularly informative connection between environmental sensing, the molecular clock and chromatin. Exposure of NRVMs to severe oxygen deprivation caused atrophy and altered expression of multiple clock-controlled genes, including induction of *Tcap*. Oxygen deprivation also changed the intracellular distribution of BMAL1 and TCAP and altered chromatin-associated histone H3.3 and H1.0. Importantly, reduced oxygen increased global chromatin accessibility and accessibility of the *Tcap* promoter, while BMAL1 depletion attenuated oxygen-deprivation-induced *Tcap* transcription. In vivo, neonatal rats maintained at 10% O_2_ similarly showed increased *Tcap* transcription and TCAP abundance. These observations are particularly interesting in light of recent evidence connecting circadian and hypoxia-sensing transcriptional mechanisms. Ruan and colleagues demonstrated that BMAL1 can directly interact and heterodimerize with HIF2A under hypoxic conditions, forming a transcriptionally active complex that regulates hypoxia-responsive gene expression and contributes to circadian variation in myocardial ischemia-reperfusion injury [10]. They identified amphiregulin (*Areg*) as a rhythmic downstream transcriptional target of the BMAL1-HIF2A complex. Arrieta et al. observe induction of *Areg* during oxygen deprivation and therefore raise the possibility that similar BMAL1-HIF2A coupling could contribute to *Tcap* regulation. Whether BMAL1-HIF2A directly occupies the *Tcap* locus under hypoxia remains unknown and represents an attractive mechanistic question for future investigation.

Perhaps the most provocative observation of the study moves in the opposite direction: from the sarcomere toward the nucleus. Arrieta et al. detected TCAP in a chromatin-enriched nuclear fraction with MYL2 also present in this compartment. Nuclear localization of TCAP was previously reported by Knöll and colleagues, who implicated TCAP in the myocardial response to biomechanical stress and demonstrated a functional relationship between TCAP and p53 under stress conditions [5]. The present work therefore does not establish nuclear localization *per se* but extends this concept by placing a fraction of TCAP within a chromatin-associated biochemical compartment.

Moreover, TCAP depletion was not transcriptionally silent. Loss of TCAP reduced basal *Bmal1* expression, modified the transcriptional response of *Myl2* to oxygen deprivation, altered fetal hypertrophic gene expression and exacerbated oxygen-deprivation-induced cardiomyocyte atrophy. Intriguingly, although *Myl2* transcription is sensitive to BMAL1 depletion under basal conditions, BMAL1 depletion did not prevent its suppression by 0% O_2_ [3]. The effect of TCAP depletion on this response therefore raises the possibility that TCAP participates in communication between environmental stress, sarcomeric gene regulation and the molecular clock rather than functioning exclusively as a downstream structural output.

These findings suggest that TCAP may not merely be an output of the circadian clock, but could participate in feedback from the sarcomere toward nuclear transcriptional networks. This interpretation should nevertheless remain cautious. Association of TCAP with a chromatin-enriched fraction does not establish direct DNA binding or chromatin regulation and altered *Bmal1* or *Myl2* expression following TCAP depletion does not distinguish a direct nuclear function from secondary consequences of perturbing sarcomeric or cellular homeostasis.

Although the authors propose that TCAP may behave as a core clock gene in cardiomyocytes, the current findings may better support a model in which TCAP is an environmentally responsive clock-controlled protein that can influence the circadian transcriptional network. Whether TCAP affects fundamental properties of the circadian clock itself remains to be established. This possibility places the findings within a broader emerging view of the sarcomere and Z-disc as signaling structures rather than passive endpoints of transcriptional regulation.

The oxygen experiments also need careful interpretation. The 0% O_2_ condition used in cultured cardiomyocytes represents severe oxygen deprivation and is distinct from the 10% O_2_ exposure used in neonatal animals. Indeed, the chromatin responses differed between the two conditions: cultured cells showed decreased chromatin-associated H3.3 and H1.0, whereas hypoxic neonatal hearts exhibited increased H1.0 without significant changes in H3.3 [3]. Establishing how physiologically relevant oxygen tensions interact with circadian regulation will therefore be important for understanding this pathway during postnatal cardiac maturation and disease.

Together, these findings raise several important questions. What determines how individual clock-controlled sarcomeric genes respond to environmental signals at the levels of phase, amplitude, transcription or protein stability? What are the secreted factors responsible for the biomass-dependent changes in hypertrophy and *Tcap* expression? Are the post-translational mechanisms previously implicated in rhythmic TCAP turnover in skeletal muscle also active in cardiomyocytes [9]? Does BMAL1-HIF2A directly regulate the *Tcap* locus during hypoxia [10]? And, perhaps most importantly, does chromatin-associated TCAP occupy defined genomic loci and directly participate in transcriptional regulation? Addressing these questions will help determine whether TCAP functions primarily as an environmentally responsive output of the cardiac clock or participates more directly in feedback between the sarcomere and nuclear transcriptional networks.

Arrieta et al. thus add an important dimension to our understanding of cardiac circadian biology. Rather than depicting the molecular clock as a timer that simply drives rhythmic expression of downstream sarcomeric genes, their results reveal a dynamic interaction between environmental signals, circadian regulation, and TCAP expression and function. In this model, the sarcomere becomes not only an endpoint of circadian regulation but also a potential participant in the cellular interpretation of time and environment.

Understanding this reciprocal communication may ultimately help explain how cardiomyocytes coordinate structural adaptation with predictable daily changes in workload and with less predictable stresses such as hypoxia or pathological hypertrophy. Whether understanding these mechanisms will ultimately inform chronotherapeutic strategies remains to be established. For now, the study raises a more fundamental possibility: in the cardiomyocyte, keeping time may require communication not only from the clock to the sarcomere, but also from the sarcomere back to the clock.

## Data Availability

There is no data associated with this research.
